# CRISPR/Cas9 Gene Editing of Gluten in Wheat to Reduce Gluten Content and Exposure—Reviewing Methods to Screen for Coeliac Safety

**DOI:** 10.3389/fnut.2020.00051

**Published:** 2020-04-24

**Authors:** Aurelie Jouanin, Luud J. W. J. Gilissen, Jan G. Schaart, Fiona J. Leigh, James Cockram, Emma J. Wallington, Lesley A. Boyd, Hetty C. van den Broeck, Ingrid M. van der Meer, A. H. P. America, Richard Gerardus Franciscus Visser, Marinus J. M. Smulders

**Affiliations:** ^1^Plant Breeding, Wageningen University and Research, Wageningen, Netherlands; ^2^John Bingham Laboratory, NIAB, Cambridge, United Kingdom; ^3^Bioscience, Wageningen University and Research, Wageningen, Netherlands

**Keywords:** gliadin, coeliac disease, ddPCR, LC-MSMS, enrichment, GlutEnSeq, T cell epitope

## Abstract

Ingestion of gluten proteins (gliadins and glutenins) from wheat, barley and rye can cause coeliac disease (CD) in genetically predisposed individuals. The only remedy is a strict and lifelong gluten-free diet. There is a growing desire for coeliac-safe, whole-grain wheat-based products, as consumption of whole-grain foods reduces the risk of chronic diseases. However, due to the large number of gluten genes and the complexity of the wheat genome, wheat that is coeliac-safe but retains baking quality cannot be produced by conventional breeding alone. CD is triggered by immunogenic epitopes, notably those present in α-, γ-, and ω-gliadins. RNA interference (RNAi) silencing has been used to down-regulate gliadin families. Recently, targeted gene editing using CRISPR/Cas9 has been applied to gliadins. These methods produce offspring with silenced, deleted, and/or edited gliadins, that overall may reduce the exposure of patients to CD epitopes. Here we review methods to efficiently screen and select the lines from gliadin gene editing programs for CD epitopes at the DNA and protein level, for baking quality, and ultimately in clinical trials. The application of gene editing for the production of coeliac-safe wheat is further considered within the context of food production and in view of current national and international regulatory frameworks.

## Introduction

Increased consumption of cereal grains has gone hand-in-hand with human development. Some 6 million years ago, as humans moved from the African forests into savannah areas, grass species with small, hard seeds became part of the human diet (1, 2). With the onset of agriculture during the Neolithic period, cereal grain consumption increased further, and has continued to do so right up to the present day. Flour milled from wheat (*Triticum aestivum* L., an allohexaploid wheat species with an AABBDD genome) became renown in Roman times for its fine viscoelastic doughs and flavorful white breads (3). Today, 220 million ha of bread wheat are cultivated annually, producing 700–750 million tons of grain annually (4), and used in a huge variety of food products (5).

Hippocrates, over 2,000 years ago, was credited with the phrase “Let food be thy medicine and medicine be thy food.” Today, whole grain foods, including wheat, that contain all parts of the grain (i.e., the bran, starchy endosperm, and the germ) are known for their health benefits, reducing the risk of several non-communicable diseases (6, 7). However, wheat consumption is also associated with the development of a variety of diseases, including allergies, auto-immune responses and non-coeliac wheat sensitivity (NCWS, also called non-coeliac gluten sensitivity, NCGS) (8–10).

The most common human disease associated with wheat is coeliac disease (CD), an autoimmune reaction prevalent in 1–2% of the global population. In genetically predisposed individuals, immunogenic epitopes, found most commonly in α-, γ-, and ω-gliadins, trigger chronic inflammation of the small intestine. These individuals carry HLA-DQ2 (>90% of the patients, mostly HLA-DQ2.5) and/or -DQ8 protein receptors on the surface of specific T cells that recognize these epitopes (11). CD leads to malnutrition and various related symptoms, ranging from bowel disorders to skin-, bone-, nerve-, and muscle-problems. CD is one of the best understood food intolerances from the perspective of human immunology and T cell specificity (12–18). The only way to prevent CD is a gluten-free (GF) diet, requiring complete exclusion of wheat, barley and rye. This is very difficult to adhere to, as gluten (especially from wheat) is added to many processed food products due to its viscoelastic and binding properties (5).

Targeted gene editing, especially CRISPR/Cas9, is a tool with considerable potential for plant development and breeding (19, 20). With the ultimate goal of removing the immunogenic gluten epitopes from the human diet, this technology is being used in the development of wheat lines with fewer gluten genes and/or gluten genes with inactivated CD epitopes. As proof of concept, CRISPR/Cas9 technology has been used to edit α-gliadin genes (21) as well as both α- and γ-gliadin genes (22–24) in bread wheat. Along with ω-gliadins, these gliadin types rank highest in abundance and overall immunogenicity compared with the low molecular weight (LMW) and high molecular weight (HMW) glutenins (11, 12). α- and ω-epitopes are highly homologous (12, 16).

Gene editing of gliadin genes will initially produce plants with a mosaic of edited, deleted, and unaffected genes. Here we discuss various methods to efficiently screen and select the most promising plant lines from a gene editing program via screens at the DNA and protein level. These selection techniques are considered in comparison with their use in screening wheat lines produced using RNA interference (RNAi), in which the transcript levels of whole groups of gliadins have been down-regulated. This has resulted in lines that have strongly reduced gluten content (25, 26). The future application of RNAi and gene editing in wheat for reduced and/or CD-hypoimmunogenic gluten will be discussed from immunological, regulatory, food technological and safety, and consumer viewpoints.

## Breeding, Genomics, Biotechnology, and Gene Editing of Gluten Genes

Bread wheat contains two groups of gluten proteins: glutenins and gliadings. Glutenins are comprised of HMW and LMW glutenins which can form a protein network and provide elasticity, and are thus essential for good bread dough quality (27–29). Gliadins (α-, γ-, and ω-gliadins) contribute viscosity to this network. The recently published reference genome assemblies of the wheat variety Chinese Spring has enabled a good estimation of the number of genes within these gene families. In Chinese Spring, 29 α-gliadins, 18 γ-gliadins, 10 ω-gliadins, 6 HMW-, and 16 LMW-glutenins have been provisionally annotated (30). In a separate study focusing on α-gliadins at the homoeologous *Gli-2* loci in Chinese Spring, 11, 26, and 10 gene copies were identified on chromosomes 6A, 6B, and 6D, respectively (31). Huo et al. (32) studied the other groups of gliadins and the LMW-glutenins on chromosomes 1A, 1B, and 1D. It should be stressed that other hexaploid bread wheat varieties may have different numbers of gluten genes (33). In addition, the expression levels during grain development can vary among these genes (31, 32) and between varieties (34).

An epitope is defined in the context of the specific human leukocyte antigen (HLA) receptor molecule (DQ2 or DQ8) that presents peptides to a T cell, as a peptide that can activate the recognizing T cell in the intolerant individual. Within gluten proteins, the epitope, a nine amino-acid peptide, is part of a larger protein fragment that is resistant to proteolytic degradation in the stomach and small intestine. Most of these resistant gluten peptides are rich in proline and glutamine amino acid residues. The glutamine residues require deamidation into glutamic acid residues at the anchor sites of the epitope for increased affinity to the HLA receptor. Sollid et al. (11) and Sollid et al. (35) have published a comprehensive list of gluten epitopes. Individual patients develop a set of T cells, each of which recognizes a different epitope (36, 37). Some epitopes are recognized by T cells in most patients (12). Considering the large number of well-documented and clinically relevant epitopes with DQ2.5 restriction, it seems justified to focus gene-editing/silencing activities on these epitopes.

Both gliadin and glutenins contain immunogenic epitopes within their protein sequences that cause CD (34, 38), but the α-, γ- and ω-gliadins contain the major and clinically most relevant epitopes (11, 12, 39–42). These are also called immunodominant epitopes, as most CD patients expressing HLA-DQ2.5 respond to the epitopes DQ2.5-glia-α1a, DQ2.5-glia-α2, DQ2.5-glia-ω1, and DQ2.5-glia-ω2, whereas most patients expressing HLA-DQ8 respond to DQ8-glia-α1 (16). There are few natural α-gliadin variants, notably on chromosome 6B, that are free of immunogenic epitopes (43, 44). So far, no food processing or classical breeding strategies have been developed that produce wheat-based food products that approach safety for CD patients (45–47), although there is a clear need to do so (48, 49).

Using chemical treatments such as ethyl-methane sulfonate (EMS) or ionizing irradiation, random mutations can be generated in the plant genome. These may result in removal of gliadin loci, as has been shown for bread wheat (23, 50). Wheat deletion lines produced with irradiation, e.g., those lacking the 6D alpha-gliadin locus with many CD epitopes (50) may be used in a breeding program, but only together with other approaches, as combining multiple chromosomal deletions is often lethal. EMS mutation breeding generates a large number of random mutations (51), it is resource-intensive, requiring extensive breeding to combine deletions in multiple gluten loci, from many plants, into one single, coeliac-safe and well-performing wheat plant (46).

**Figure 1:**
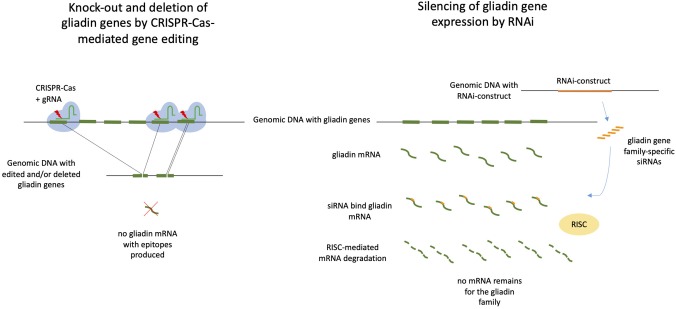
CRISPR/Cas9-mediated gene editing and deleting vs. RNAi-mediated silencing of gliadin genes in wheat. Both approaches may be used to prevent that gliadin proteins with Coeliac disease epitopes are being produced. On the left: CRISPR/Cas9 with gliadin-specific gRNAs target gliadins genes resulting in loss of genomic fragments containing full gliadin genes and/or small internal deletions in the gliadin genes. Both deletions may lead to knock-out of gliadin gene expression. The ultimate mutant wheat plants are free of CRISPR/Cas9 constructs. On the right: A RNAi construct targeting gliadin genes that has been inserted in the wheat genomic DNA produces small interfering RNAs (siRNAs), which bind to gliadin mRNA. The complex of mRNA and siRNA is recognized by the plant RNA-induced silencing complex (RISC), resulting of degradation of mRNA and silencing of gliadin gene expression. A RNAi construct targets one of the gliadin gene families.

Two modern biotechnology approaches represent promising tools toward producing wheat that is safe for CD patients: RNA interference (RNAi) and CRISPR/Cas9 gene editing (Figure 1). Using RNAi, Gil Humanes et al. (52) targeted γ-gliadins, while Becker et al. (53) aimed to silence α-gliadin genes. Gil-Humanes et al. (25) used RNAi to down-regulate all three gliadin gene types resulting in an up to 92% reduction in the gliadin response, as estimated using the R5 monoclonal antibody (mAb) assay, and a 10–100-fold reduction of DQ2 and DQ8 epitopes in T-cell tests. More recently, Altenbach et al. (54) used RNAi to silence ω-gliadins. However, implementation of these methods faces several hurdles. RNAi targets gluten genes indirectly (through their RNA transcripts) and this approach requires stable genetic modification (GM) and transgene expression. Governmental regulations for GM food products require expensive and time-consuming food-safety assessments to be met before product marketing.

The recently developed CRISPR/Cas9 gene editing system has the potential to simultaneously and precisely modify multiple gliadin-encoded epitopes, and/or delete some of the genes, while potentially maintaining the food-technological quality of the gliadin proteins. Application of CRISPR/Cas9 has been successful for targeted mutagenesis of single copy genes across all three homoeologous loci in hexaploid wheat (55). However, mutating target-specific sequences in large gene families in a polyploid species, such as gluten-encoding genes in wheat, has only recently been reported (21–23).

In CRISPR/Cas9 gene editing, a single guide RNA (sgRNA) directs the Cas9 endonuclease to the target DNA sites where it creates a double-strand break. During repair, the innate DNA repair mechanism of the plant can generate mistakes, usually resulting in small deletions of one or a few nucleotides. In the case of the tandemly repeated gliadin genes in wheat, simultaneous double-strand breaks may occur in consecutive genes, which can result in deletion of large DNA fragments carrying one or more gliadin genes (23). Large deletions between multiple target sites have also been obtained, for example in rice (56).

## Analytical Methods to Characterize CRISPR/Cas9-Induced Mutants

The random occurrence of deletions at the target sites, and the large number of targets in the gliadin gene families, means that gene editing will initially produce plants with a mosaic of edited, deleted and unaffected genes. This requires efficient methods to detect the plants with edits, in order to be able to rigorously reduce the number of plants in a program through quality-directed selection steps. Screening may occur at the DNA level (the number of genes present and their sequences after editing), at the protein level or for baking quality and immunity. The same methods can also be used to characterize RNAi lines, with the exception of screening at the DNA level, as RNAi interferes with the mRNAs after transcription, while the gluten genes themselves remain intact. These methods may be combined into a screening pipeline.

In Table 1, we summarized the screening methods that have been used in the RNAi and CRISPR/Cas9 studies on gluten in wheat published to date. We describe these methods below.

**Table 1:** Methods used for screening wheat lines produced using RNA interference or gene editing using CRISPR/Cas9.

**Technology type**	**Aim**	**Applied by**							
		(52)	(25)	(53)	(57)	(21)	(22)	(54)	(58)
**RNAi:**		*	*	*	*			*	*
**CRISPR/Cas9:**						*	*		
	Target	A high-transcript-level 169 bp sequence targeting most γ-gliadins; testing RNAi technology	Three plasmid sequences (all with 361 bp chimeric conserved fragments) and their combinations to targeting all α-, γ-, and ω-gliadins	A 313 bp conserved α-gliadin fragment targeting all α-gliadins	Seven plasmid sequence combinations targeting all α-, γ-, and ω-gliadins and LMW glutenin	Two sgRNAs targeting immuno-dominant 33-mer α-gliadin	Six sgRNAs targeting α- and γ-gliadin signal peptide and/or two epitope regions	A 141 bp ω-gliadin fragment targeting ω-1,2 gliadin	A 217 bp fragment with three targets from D-genome α-gliadin genes, targeting α-gliadins
	Transformation	Particle bombardment of scutellum tissue of two bread wheat lines; D-Hordein promoter for hpRNAs expression; *bar* for PPT selection	Gold particle bombardment of immature scutellum tissue of two bread wheat lines; endosperm-specific promoter; *bar* for PPT-selection	Particle bombardment of immature embryos from a single cultivar; CaMV 35S promoter; *nptII* gene for kanamycin resistance	Gold particle bombardment of immature scutellum tissue of one bread wheat cultivar; D-Hordein promoter for hpRNAs expression; *bar* for PPT-selection	Gold particle transformation of scutellum tissue of 2 bread wheat lines and 1 durum wheat cultivar; Ubiquitin 1 promoter from maize; *bar* for PPT-selection	*Agrobacterium* transformation of one bread wheat cultivar; *ACTIN* promoter; *nptII* for G418-selection	Gold particle bombardment of young embryo-derived callus from a single bread wheat cultivar; HMW-GS Dy10 promoter; *bar* for PPT-selection	Gold particle bombardment of young embryo-derived callus from Butte 86; maize *Ubi1* promoter; *bar* for PPT-selection
**DNA**									
PCR	Confirmation of presence of transgene in T0 plants	+	+	+ (RT-PCR for RNAi construct expression testing)	+	+	+ (presence of Cas9 and all sgRNAs; TR-PCR for expression of Cas9)	+	+
Illumina HTP DNA sequencing	Indel characterization and quantification	NA	NA	NA	NA	+	+ (applied after gene-enrichment in GlutEnSeq (below)	NA	NA
Sanger sequencing	Off-target mutation	NA	NA	NA	NA	+ (but not detected)	–	NA	NA
ddPCR	HTP gene copy number (variation) assessment	NA	NA	NA	NA	–	+ (duplex ddPCR includes reference comparison)	NA	NA
GlutEnSeq	Gene enrichment; Identification of large- and medium-sized mutations/deletions	NA	NA	NA	NA	_	+	NA	NA
**PROTEIN**									
Acid-PAGE; SDS-PAGE	Gluten profile analysis	+ (Acid-PAGE)	+ (Acid-PAGE)	+ (SDS-PAGE)	+ (Acid-PAGE of T1 seeds for homozygosity testing; Acid-PAGE and SDS-PAGE of T3)	+	+ (Acid-PAGE)	+ (SDS-PAGE)	+ (SDS-PAGE)
2-DE (2D gel-electrophoresis)	Intensity measurement of specific gluten proteins (in T1 compared with original plant)	–	–	+	–	–	–	+	+
MALDI-TOF	Confirmation of PAGE gluten profiles	+ (MALDI-TOF MS)	–	+ (MALDI-TOF MS for analysis of individual spots from 2-DE)	–	+	–	–	–
HPLC	Quantification and characterization of individual gliadin and glutenin protein groups	–	+ (RP-HPLC)	+ (RP-HPLC)	+ (RP-HPLC)	+ (RP-HPLC)	–	–	–
nanoLC-MSMS	Identification of peptide fragment spectra to be matched to protein sequences in database, and comparison to control	–	–	–	+ (LC-MS/MS)	–	+ (measuring protein reduction and compensatory effects)	+ (MS/MS of isolated 2-DE spots from control plant (to confirm absence of target protein in transformed lines)	+ (MS/MS of isolated 2-DE spots from control plant (to confirm absence of target protein in transformed lines)
**BREAD QUALITY**									
SDS sedimentation	Measuring gluten strength for prediction of processing and end-product qualities	–	+	–	+	+	–	+	+
Mixing properties	Assessment of dough resistance, development and stability	–	–	–	–	–	–	+ (Mixograph)	+ (Mixograph)
Rheology testing	Maximum resistance of dough to extension (RE) and extensibility (EX)	–	–	+	–	–	–	–	–
**IMMUNE RESPONSE**									
Monoclonal antibodies	Total gluten content in food; 33-mer is target peptide for G12	+ (R5 for total gliadin content)	+ (R5 for total gliadin content)	–	G12 (total gluten content)	+ R5 and G12 (gluten content and impact on 33-mer)	–	–	–
Serum reactivity of CD patients	Immunogenic potential of transgenic lines	–	–	–	–	–	–	+ (IgG and IgA antibody reactivity)	+ (IgG and IgA antibody reactivity)
T cell proliferation response	Testing epitope-specific reactivity	–	+ (TG2-treated protease-digested total gluten extract)	–	–	–	–	–	–
Food challenge	Coeliac food safety assessment	–	–	–	–	–	–	–	–
**OTHER ASPECTS RELATED TO MUTANT/TRANSFORMED PLANT PERFORMANCE**									
Mutation/transgene stability in T generations		+ (measured in T3)	+ (aiming at homozygosity)	+ (stable integration and expression in T2)	+	+	–	–	–
Absence of transgenes in T generations		NA	NA	NA (3–11 transgene copy numbers in transgenic plant lines)	NA	+	–	NA	NA
Changes in expression of other gene families		–	–	+ (RP-HPLC analysis of gluten protein profile: no changes in γ- and ω-gliadin profiles)	–	+ (analysis of γ- and ω-gliadin, LMW and whole bread wheat genome by Sanger sequencing)	–	–	+ (silencing of some HMW glutenins)
Chromosome number		–	–	–	–	+	–	–	–
Performance (growth; fertility; seed quality/quantity)		+ (full fertility; normal grain morphology and weight)	+	+ (full fertility; normal seed set and grain morphology;	+ (fertility; days to anthesis normal; kernel weight differences)	+	–	+ (fertility; normal kernel weight)	+ (fertility; normal kernel weight)

*NA, not applicable*.

### Screening for Edits in Gliadin Genes at the DNA Level

The hexaploid bread wheat genome is composed of three diploid sub-genomes (2n = 6x = AABBDD), is ~17 Gb in size, and contains a large number of repeat sequences (59, 60). Consequently, sequencing the whole genome of edited lines is not currently feasible and a complexity reduction step is necessary. RNA-seq would reduce complexity, but will not reveal all gluten genes, as not all genes are expressed and gene expression is developmentally and/or environmentally regulated (34). In addition, the immature T1 seeds in which the genes are being analyzed are not available for destructive sampling. Therefore, leaf DNA samples of T0, T1, or later generations are usually sampled. Amplicon sequencing of the target region, or a complexity reduction step on the genomic DNA described below, may then be applied.

#### Amplicon Sequencing

In the study by Sánchez-León et al. (21), 47 T1 lines were selected and the CRISPR/Cas9-induced mutation was confirmed by amplifying the target regions using polymerase chain reaction (PCR), followed by Illumina MiSeq sequencing of the amplicons using 2 × 280 bp paired-end reads.

#### GlutEnSeq Enrichment

A gene enrichment exome-capture methodology was developed by Jouanin et al. (23) to pull down all gluten genes from the wheat genome, thereby reducing DNA complexity before sequencing (61). Termed GlutEnSeq, this approach uses probes that represent all known sequence variations present in thousands of available prolamin gene sequences from numerous *Triticum* species and varieties (22). The in-solution enrichment system was validated with cv. Chinese Spring deletion lines [(62); a panel of lines, containing deletions spanning defined regions of single chromosomes] and γ-irradiated lines of cultivar Paragon (Wheat Genetic Improvement Network, UK). Pulled-down gluten genes were sequenced using Illumina MiSeq 2 × 250 bp paired-end reads and analyzed against the Chinese Spring RefSeq v1.0 reference genome (63). Analysis of the sequence revealed that gluten gene sequences were generally enriched around 10,000-fold. The coverage profile of the cv. Fielder CRISPR/Cas9 gliadin mutant lines revealed examples of a homozygous deletion of the γ-gliadin *Gli-1* locus on chromosome 1B and a heterozygous deletion of the α-gliadin *Gli-2* locus on chromosome 6A (23). Further bioinformatic analyses of the sequence reads is still required to determine how effective GlutEnSeq is at detecting small insertion/deletions (indels) at the CRISPR/Cas9 target site in gliadin genes and thus the efficacy in a large screening program.

#### Droplet Digital PCR (ddPCR)

An alternative approach is to analyze the number of genes that remain, before proceeding to a sequencing step. ddPCR is a PCR-based assay in which prior to PCR amplification, DNA fragments are partitioned into typically 20,000 water-in-oil droplets. This enables multiple, simultaneous end-point amplification reactions from a single sample with high quantitative precision and reliability. It is a reliable method for high-throughput gene copy number quantification (64), which is relevant for multiple sgRNA CRISPR/Cas9 editing where targeting α-gliadins and/or γ-gliadins (23, 24), as this results in plants with different copy number variations (CNV) of the target genes. The accuracy and reliability of ddPCR has been determined for transgene copy number analysis in crops, including wheat (65). Jouanin et al. (24) optimized the method for CRISPR/Cas9-induced mutations in gliadin genes in wheat, and developed duplex ddPCR assays where one part of the duplex assay was directed to a single copy reference gene on each of the six homoeologous wheat chromatids, and the other assay to the target regions in the gliadin genes. The method was validated using selected Chinese Spring deletion lines, Chinese Spring nullisomic/tetrasomic lines [(66); in which one entire chromosome pair is deleted, balanced by an extra pair of the remaining homoeologous chromosomes], Paragon γ-irradiated lines (which had displayed changes in Acid-PAGE gluten protein patterns) and synthetic hexaploid wheat accessions (alongside their component parent species *T. durum* and *Aegilops tauschii*) (24).

### Screening for Edits in Gliadin Genes at the Protein Level

Acid-PAGE is a well-known, reliable, non-denaturing gel electrophoresis technique used to differentiate and identify wheat varieties based on their gliadin protein profile. Separation is based on protein molecular weight and charge (67). In RNAi lines, Acid-PAGE clearly showed the down-regulation of groups of protein bands related to gliadin gene families. In gene-edited lines it revealed band position changes, as well as band absences, when compared to the wild type (WT) (22). Acid-PAGE or SDS-PAGE has been used in all RNAi and CRISPR/Cas9 studies of gliadin genes published to date (Table 1). However, one-dimensional PAGE does not give full information on gluten proteins, since the protein spots overlap, as is visible in 2D-electrophoresis (68–73). Changes in the Acid-PAGE or SDS-PAGE one-dimensional gel electrophoresis profiles should therefore be confirmed by proteomic techniques. Several targeted approaches for quantitative analysis of gliadin and glutenin proteins have been described (74–77).

To characterize the changes in expressed proteins in gene-edited wheat lines, various proteomics methods have been employed. The choice of method strongly depends on the available equipment. With regard to the approach, non-targeted proteomics analysis of the prolamin fraction would be most appropriate for situations with novel changes in the gluten proteome. However, proteomics is complicated for this set of gene families, and a complete description of the peptidome generated by wheat digestion is complex (78). The identification of proteins in a non-targeted proteomics analysis depends to a large extend on the availability of the protein sequence information (79). However, in particular, gene editing may generate novel peptides that are not present in any library and can only be predicted by sequencing all gluten genes in each gene-edited line. Most studies use a targeted approach, focusing on a small set of peptides as reference for a class of gluten proteins (76, 77, 80). The extraction procedure used is also relevant, as it has impact on proteins and peptides recovered (81, 82).

#### Matrix-Assisted Laser Desorption/Ionization With Time-of-Flight Mass Spectrometer (MALDI-TOF)

Gil Humanes et al. (52) used MALDI-TOF to identify and quantify peptides derived from glutenins and gliadins, as a method to quantify the amount of those proteins present in grains of their RNAi lines.

#### High-performance liquid chromatography (HPLC)

Sánchez-León et al. (21) used reversed-phase high-performance liquid chromatography (RP-HPLC) to quantify glutenins and gliadins in half-seeds of CRISPR/Cas9 lines.

#### HPLC and Mass Spectrometry (MS)

García-Molina et al. (48) studied the impact of silencing of RNAi lines on both target and on non-target proteins. They performed separate analyses on four kernel protein fractions, including gliadins and glutenin fractions, by combining a 2D electrophoresis gel analysis with RP-HPLC and nano-electrospray ionization mass spectrometry (nESI-MS/MS) of individual protein spots excised from the gels. Liquid chromatography-tandem mass spectrometry (LC/MS/MS) data were used as queries in searches against UniProt and TrEMBL databases.

For a detailed review on the use of proteomics methodology as a tool for screening for immunogenic peptides in cereals we refer to Alves et al. (79).

### Bread Quality of Gliadin Gene Edited Wheat Lines

Wheat with less gluten, or with gluten that does not contain CD epitopes, needs to retain baking quality to be acceptable to producers and consumers as new gluten-free products (46). SDS sedimentation, mixing properties and rheology testing can all be performed on a small scale, but require grain of fixed-edit lines to be multiplied. For example, 10 g of seed are needed for a dough extensibility test using a Micro-Farinograph (83, 84). That means that one or two extra rounds of seed multiplication must be carried out before dough properties can be studied. Most studies in Table 1 have determined SDS sedimentation volumes, but only (53, 54, 58) tested rheology, as well as (85).

### Immune Response of Gliadin Gene Edited Wheat Lines

Kits based on the monoclonal antibodies (mAbs) R5 or G12 are routinely used to measure the amount of gluten in foodstuffs, as they recognize short peptides that are present in many gliadins. They have used to measure the decrease in gluten content in RNAi lines (Table 1). However, they are not accurate when using in CRISPR/Cas9 lines if the target sequence edited are the CD epitopes. This is because as the mAb recognition site is much shorter than the nine amino acid epitopes recognized by the CD-immunologically relevant human T cells, a single amino acid replacement in the epitope induced by gene editing maybe sufficient to abolish binding (15, 38), but may go unnoticed when screening with mAbs.

Human T cells that recognize gluten epitopes have been cultured and they can be used for assays, but the reaction is very specific, and qualitative rather than quantitative (38, 86). The T cells will only proliferate when the 9-amino acid peptide that they recognize is present (usually after deamidation). Each patient has many T cell clones that recognize CD epitopes, but across patients there are T cells that recognize the same, “major” epitopes (12), and gliadins with these epitopes are the main target of gene editing. The major epitopes are listed in Sollid et al. (11) and Sollid et al. (35). T cell tests have been performed in a few of the studies in Table 1.

### Clinical Trials

Clinical trials with food challenges are meaningful once the other tests indicate that the level of major CD epitopes is very low. Serum reactivity and T cell proliferation responses have been measured in some studies. Larger amounts of wheat flour are required in order to bake bread for human food challenges. Gil-Humanes et al. (87, 88) produced and tested bread using wheat flour from an RNAi line with very low content of gliadins and acceptable baking and sensory properties. The bread has been tested for safety in rats (89) and subsequently it has been used in a food challenge with people with NCWS (90). Challenged and control people both did not show gluten immunogenic peptides in their stools and responded similarly in a Gastrointestinal Symptom Rating Scale (GSRS) questionnaire. Consumption of low-gliadin bread increased butyrate-producing bacteria and favored a microbial profile that may improve gut quality (90).

## Combining Methods Into Pipelines to Efficiently Screen CRISPR/Cas9 Wheat Mutant Lines

There are many tens of gluten CRISPR/Cas9 gene targets in the wheat genome. Each regenerated plant (T0 generation) from a single gene editing experiment may be a mosaic of deleted, modified and unchanged genes. Initially the edits may be present in heterozygous form. Analysis of T1 seeds revealed that of about 50 different highly presented α-gliadin sequences in bread wheat and durum wheat, 25–78% were mutated by CRISPR/Cas9 as determined by Illumina sequencing (21), while Acid-PAGE analysis of CRISPR/Cas9 derived germplasm has found up to 30% of the proteins had changed (22). In the next generation of self-pollinated transformed plants, segregation of heterozygous edits will occur. The numbers of seeds to be analyzed will increase considerably; from each T1 wheat seed a plant with 5 ears, each containing 50 seeds, can develop. Efficient analysis of high numbers of progeny seeds becomes relevant: hundreds of seeds may be required to be screened from tens of T0 transformants, and a fast method for pre-screening is therefore required. The T1 generation in the study of Sánchez-León et al. (21) consisted of 6-12 mature T1 grains from each of the 21 T0 plants obtained, so in the order of 200 plants. Jouanin et al. (22) tested 1,149 T1 seeds obtained from 117 regenerated T0 plants.

**Figure 2:**
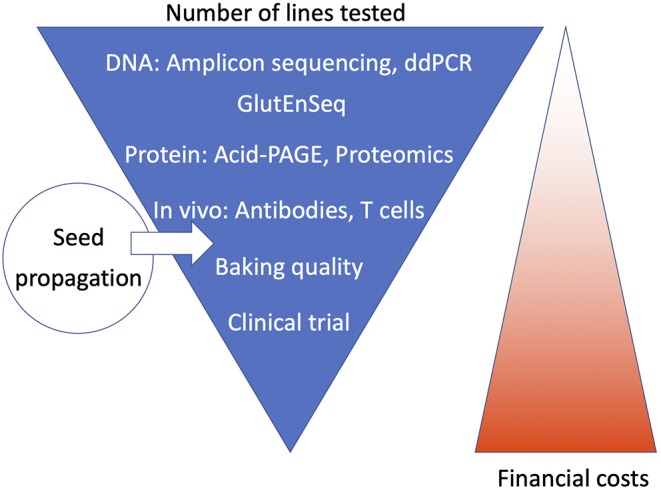
Schematic overview of how the steps in the analysis pipeline may be ordered. Ideally, the first and second step use fast, high throughput, and cheap methods that screen DNA or gluten proteins for the presence of changes. Subsequently, more precise but slower and more expensive methods can be employed to precisely characterize the remaining lines, culminating in tests for baking quality and clinical trials, for which a seed propagation step would first be required to produce sufficient amounts of grain.

For an analysis pipeline to be applicable for high-throughput screening, ideally it should reduce the number of lines to be tested at the earliest possible stage, i.e., in the first and second step. The pipeline should employ cheap or fast screening methods to detect the presence of differences in DNA of gluten protein, without the need for precise characterization, as that may be done subsequently on a much-reduced number of lines. We summarize this approach schematically in Figure 2, and below we describe the steps used in two examples of screening pipelines that have been applied in recent CRISPR/Cas studies. A general description of the relative advantages and disadvantages of each of the methods in terms of accuracy, time requirement and cost effectiveness cannot be given, as this depends very much on the equipment and experience present in the laboratory. For instance, while some labs may have used acid PAGE routinely, others may not have done so and may therefore prefer SDS-PAGE. Not all labs have a ddPCR machine. Proteomics methods strongly depend on the availability of highly specialized equipment.

### The α-Gliadin 33-Mer Target Approach

Sánchez-León et al. (21) focused on a series of analytical techniques to make an in-depth evaluation of gene-edited events over three generations (T0 to T3). This was to understand the effects and efficacy of CRISPR/Cas9 toward a coeliac-safe end product. Their pipeline was therefore not primarily intended to be a sequential-selection pipeline designed to reduce the numbers of mutant lines and retain only the “most promising” mutants. The pipeline consisted of four steps:

1. Illumina DNA sequencing to characterize the DNA extracted from leaves harvested from T1 transgenic plants and the corresponding wild-types to measure the frequency and types of indels. Deletions up to 126 bp and insertions of up to 158 bp were found. Remarkably, most of the deletions in the target region (α-gliadins possessing the immuno-dominant epitope-containing 33-mer peptide fragment) were multiples of 3 bp; deletions of −3, −9, −36, and −78 bp were observed at high frequencies in the three selected T1 mutant lines from the reported transformed bread wheat cultivar. High frequencies of frameshift-inducing −1 and +1 bp (typical for CRISPR/Cas9 mutations) were, however, not found.
2. Subsequently, to assess the impact of the observed mutations on seed gluten protein composition, T1 seeds of several tens of plants each from five selected T0 plants were assessed qualitatively for protein composition changes using Acid- and SDS-PAGE, and further quantitatively analyzed by MALDI-TOF and confirmed by HPLC. The mutant lines reported showed reduced total gliadin content (specifically the α-gliadins), increased glutenin content (especially HMW) due to a compensatory effect and, as a result, a lower gliadin/glutenin ratio.
3. Confirmation of significantly reduced α-gliadin content in mutant lines was followed by investigation of any reduction in immune reactivity, for which T2 seeds were analyzed with R5 and G12 monoclonal antibodies. An average reduction of over 60% total gluten was found, with an 85% reduction in gluten content observed in one line.
4. Finally, bread-making quality of mutant lines was assessed using the SDS sedimentation test. Using flour produced from bulked T2 and T3 seeds of several mutant lines, SDS sedimentation results predicted reasonably good quality and bread-making performance for the edited lines.

While this pipeline identified and characterized mutations in gliadins, there are a number of other issues that were considered: (a) CRISPR/Cas9 mutagenesis may create off-target mutations. Accordingly, Sánchez-León et al. (21) carried out Sanger sequencing of cloned α-, γ-, and ω-gliadin genes and of the entire wheat genome (with specific focus on loci containing the presence of potential off-target sites) from mutant lines and were able to conclude that no off-target mutations had occurred. This demonstrated the high specificity of the chosen sgRNAs. (b) Another issue relates to the transmission of the mutations to subsequent generations. Via RP-HPLC and Illumina sequencing, the inheritance of the gliadin and glutenin profile (prolamin phenotype) was confirmed. Interestingly, presence or absence of the Cas9 expression vector in T2 plants did not appear to affect mutation frequencies compared to the original T0 plant. (c) PCR and Illumina sequencing were also used to test whether any of the gene-edited low-gluten wheat lines lacked the transgene and insertions at the cleavage site. Three bread wheat and six durum wheat lines were identified as transgene-free and insertion-free. These lines were fully fertile, set seeds and had normal chromosome numbers. (d) The resulting low-gluten, transgene construct-free wheat lines may provide useful source material to introgress the low-gluten/low-immunogenic trait into elite wheat varieties, or to undertake further iterative CRISPR/Cas9-mediated improvement.

### α- and γ-Gliadin Multiple Target Approach

Jouanin et al. (22) edited α- and γ-gliadins with multiple gsRNAs in a single construct. Notably, α-gliadins are present as tandem repeats on the group 6 chromosomes. Therefore, it is possible that repair of simultaneous double-strand breaks in such regions could lead not only to small deletions but also to “allele drop out.” The optimized pipeline for screening of the plants produced would consist of the following steps.

1. DNA is extracted from young T1 leaves and analyzed using ddPCR for changes in gene copy number. ddPCR can measure whether gliadin genes are deleted compared to the number present in the original Fielder plant (24).
2. Proteins are extracted from the endosperm of selected “promising” T1 seeds for Acid-PAGE analysis to look for the corresponding changes in protein profiles, i.e., gliadin bands that are missing compared to the original plant (22).
3. Leaf DNA from the best candidate T1 lines is then used to perform GlutEnSeq (23). The captured gluten genes are sequenced and compared to the corresponding gluten genes pulled down from the original variety. In this way changes in the DNA sequence of gluten genes can be identified.
4. The most interesting edited wheat lines from the GlutEnSeq analysis are self-pollinated and the resulting T2 or T3 seeds analyzed at the protein level using LC-MSMS.

Once identified, gene-edited lines that are complementary based on the types and numbers of eliminated or inactivated coeliac-immunogenic epitopes they carry, can be crossed to combine the gene-edited gliadins. The progeny should also be screened to select against inheritance of the CRISPR/Cas9 construct. The resulting genetically stable plants can be analyzed and selected following the pipeline above, up to the LC-MSMS step. GlutEnSeq enrichment followed by MiSeq sequencing of lines selected using this pipeline will enable the gsRNAs to be fine-tuned to those remaining genes that need to edited. Subsequent analysis via LC-MSMS should be limited to the most-promising lines only, due to the complexity and labor costs of the analysis. Ultimately, lines with multiple gene-edited gliadins would require advanced proteomics analysis (see above), followed by immunological assays (including epitope-specific mAbs or T cells) whereby the flour from new epitope-mutant wheat lines would be screened with panels of T cell clones to test for a CD-hypoimmunogenic reaction. Ultimately confirmation through human intervention studies, involving analysis of biopsies from the small intestine or analysis of IgA anti-TG2 antibody levels in serum, would be needed.

## Technical Considerations for CD-Hypoimmunogenic Gluten

Up to now, the use of RNAi has resulted in the E82 line with strongly reduced levels of gliadins, that has been tested on people with NCWS (90). This line had good dough quality while the gliadin level was strongly reduced.

With CRISPR/Cas9 it is possible to edit all gliadin epitopes by causing local deletions and frameshifts. However, after the first round of edits, modifying the few remaining epitopes in some of the genes would require multiple rounds of additional edits. Specific amino acid substitutions in a gliadin epitope can abolish its immunogenicity (38) whilst having no effect on gene expression and thus on bread dough quality. With new developments in gene editing being published almost monthly, these options may become available soon, meaning that the goal of editing the wheat gluten genes at the epitope level into CD-safe wheat lines may be realized (26). Base editors are very promising. For this, CRISPR systems have been developed with a deactivated Cas9 (dCas9) or a Cas9 single strand nuclease (nickase) fused to a second active enzyme such as cytidine deaminase. The sgRNA directs the dCas9-cytidine deaminase to the target site, to enable deamination of a target cytosine into a uracil, which is subsequently converted to a thymine through DNA replication and repair. This approach has recently proven successful in wheat (91). Adenine deaminase-based DNA base editors have also been developed recently to extend the range of amenable target sites [reviewed in e.g., Eid et al., (92)].

In theory, an alternative to base editing is gene correction or gene replacement (93, 94). Techniques for this do not yet have the efficiency required for editing a gene family. It would be promising for template-based targeted replacement of CD-immunogenic genes by safe gene variants from the same accession or variety. For instance, Van Herpen et al. (43) identified some presumably safe α-gliadin genes at the *Gli-2* locus on chromosome 6B. Duplication of these genes through gene replacement at the loci on 6A and 6D would efficiently remove many immunogenic epitopes. Prime editing (95) would be a suitable technique for this goal. The screening of plants produced by this approach would be relatively straightforward, as the desired changes are defined beforehand. This could offer the safest approach, as the replaced genes would be exact copies of existing genes that are considered safe.

These alternatives make it more feasible to maintain the complete gluten proteome, while removing CD-epitopes. This may be advantageous, because removal of gluten loci may trigger protein compensation due to overexpression of remaining gluten genes (96), which may promote other CD-immunogenic epitopes, and would negatively affect bread dough rheology and other food technological characteristics as well. Compensation effects by increased production of ω-gliadins, which also have CD epitopes, have for instance been found in the CRISPR/Cas9 α-gliadin edited wheat lines of Sánchez-León et al. (21).

As CRISPR/Cas9-induced deletions and the alternative strategies above present advantages, inconveniences and uncertainties for editing gliadin genes toward wheat with hypoimmunogenic gluten, one strategy would be to combine several of these methods sequentially to generate the safest wheat variety for CD-patients while retaining sufficient baking quality. As an example, in a single wheat plant, the α-gliadin gene family (having the least diversity and being the best characterized) could have their immunogenic epitopes replaced by known safe ones using the Cas9/template approach, while the more complex γ- and ω-gliadin genes families on the group 1 chromosomes could be deleted. As a result, no CD-immunogenic gliadin epitopes would remain but CD-safe α-gliadin proteins would still be produced which retained the viscosity characteristics necessary for bread dough quality.

Finally, rather than relying on modified gliadins that are probably safe, the safest approach would be to design gliadins that are absolutely inert by avoiding the spacing of specific amino acids that enable gliadin peptides to fit into the groove of the HLA-DQ2.2–T cell, HLA-DQ2.5–T cell or the HLA-DQ8–T cell complexes, and insert these in lines without native gluten genes. These genes can be designed, as the structural requirements of these complexes have completely been elucidated (13–16, 97–99) and they can be introduced into the DNA replacing toxic gluten genes using newly developed tools such as Prime editing (95).

## Issues Regarding the Future of Gene Editing of Wheat for CD-Hypoimmunogenic Gluten

### Immunology

In theory, CRISPR/Cas9 editing could result in the generation of peptide sequences that have the potential to become new epitopes. However, newly formed epitopes that are clinically relevant are likely to be similar in amino acid sequence order to existing epitopes and thus could be screened out. In addition, the flour from new, epitope-mutant wheat lines would be screened with panels of T cell clones to test for a CD-hypoimmunogenic reaction. As a final precaution, testing at the pilot product level with volunteer patients would need to be carried out prior to market introduction. With the current improvements and fine-tuning of gene-editing techniques close at hand, it is realistic to face the future positively and to embrace the opportunities offered by gene-editing. Monitoring of possible negative, clinically-relevant effects after large-scale market introduction of gene-edited wheat should however be standard.

### Regulation Concerning GM

RNAi requires foreign DNA (with inverted copies of a piece of the target gene(s)) to be present in plants. Gene editing requires foreign DNA (constructs encoding zinc finger proteins, TALENs, or Cas9 plus guide RNAs in case of CRISPR/Cas9) or proteins to be introduced in plant genomes or plant cells, as a transient step to enable editing of the target genes. Foreign DNA will be segregated out in subsequent generations, so that they are not present in the final gene-edited lines and the end products. In an alternative approach, Cas9 protein plus guide RNAs are introduced into cells, avoiding transformation and subsequent need for segregation to remove the construct. The issue which needs to be addressed is whether to regulate gene-edited plants—and derived products—according to a process-based or a product-based approach. A process-based approach would consider that induced mutations would fall under GM regulations, even if foreign DNA or proteins were not present in the final plant. Conversely, a product-based approach would consider the absence of foreign DNA or protein in the final gene-edited plant as similar to traditional breeding, where the presence of similar mutations in plants obtained through spontaneous or mutation breeding e.g., through use of γ-irradiation or EMS, are exempted from GM regulations. Such mutation breeding approaches follow conventional breeding rules because of a history of “safe use,” and have been exempted from EU regulation by putting them on Annex 1B of the GMO Directive 2001/18/EC (100).

On July 25th 2018 the European Court of Justice ruled that, according to the text of the Directive 2001/18/EC (100), plants produced with gene editing as a mutation technique are not exempted from GM regulation as long as it has not been “conventionally used” in “a number of applications” and have “a long safety record” (101, 102). In contrast, many other countries have chosen not to regulate plants produced with gene editing as GM, provided no foreign DNA is present in the final product. Canada will evaluate them within their existing framework for Plants with Novel Traits. Consequently, gene-edited wheat lines with hypoimmunogenic gluten and derived products can be developed and widely commercialized, but they will not be accepted in the EU without fulfilling costly GM-related tests and labeling [up to 100 million euro per case; (103)]. This decision will have serious consequences within the EU regarding the application of the technique, for companies with regard to the production of CD-safe wheat varieties and derived food products, and for CD-patients with regard to the availability of such safe foods (104, 105).

### Public Acceptance

It has been shown that people suffering from food-related disorders are usually positive about the development of healthier products for their disorder. This positive attitude has also been observed in non-patients regarding the personal benefit of health-safe and health-promoting application of GM technologies (106, 107). For patient societies, the methods used to produce safe food are not an issue. Their concern relates to proper testing and labeling, so that coeliacs can distinguish gluten-free wheat or wheat with safe gluten from “normal” wheat, which will lead to stricter regulation of food packaging and ingredient information. If there is ultimately success in producing wheat varieties with acceptable levels of gluten through gene editing techniques, it will be of interest to see whether such a valuable trait can contribute to a change of heart and mind of the general public toward the application of biotechnology for safe food.

### Gluten-Free Food Labeling

Currently, products labeled as gluten-free have to contain <20 ppm of gluten (108), which is assessed using R5-ELISA (R-Biopharm, Darmstadt, Germany), the recommended type I method according to the Codex Alimentarius Commission (109) and Bruins Slot et al. (110). However, in the case of analyzing products made of hypoimmunogenic wheat/gluten, measuring the total gluten content of the product is no longer relevant. Rather, it is the total amount of immunogenic peptides that is relevant. Since monoclonal antibodies recognize only a maximum of five or six amino acid sequences within a protein, they are unable to detect complete 9-amino-acid long T cell epitopes, nor will they be able to distinguish intact epitopes from epitopes with one or two amino acid replacements, even though this may be sufficient to abolish immunogenicity (38). Therefore, proteomics techniques are required for the assessment of gluten status. A first step toward this has been made: a mass spectrometric (LC-MRM/MS) method has been developed to detect quantitatively and simultaneously a set of specific CD-epitopes at the femtomolar detection level in wheat seed extracts in a high-throughput manner (74, 78, 111).

### Food Technology

An intermediate goal is wheat lines with greatly reduced epitope numbers related for some or all gliadin families. These lines may not be sufficiently safe for sensitive CD patients, but they would reduce the induction of the disease in susceptible people (children, as well as adults) since gluten dosage correlates with the induction of the disease (112).

### Production Chain Requirements

When hypoimmunogenic wheat varieties make it to the market, whether made by gene editing, RNAi, alone or in combination with other approaches [such as low prolamin mutations (113)], it will initially require a separate production chain, implying completely separated facilities for hypoimmunogenic wheat on farms, at the processing factory, and all the way to packaging and labeling, to avoid any risks of contamination with other grains containing immunogenic gluten epitopes. If such products are well-accepted and the market grows, consumers may prefer safe wheat over regular wheat, even if they are not coeliac patients themselves, so more products will be made with hypoimmunogenic gluten. At some point they may be considered as the new standard for gluten. In that case, the main production chain in, for example, a region, could become gluten-safe. In the long run, this trend could expand, replacing regular wheat varieties by hypoimmunogenic ones, and abolishing the need for separate production chains—although strict precautions should remain in place to avoid contamination with related species that contain immunogenic gluten epitopes.

## Conclusions

Gene editing using CRISPR/Cas9 offers the prospect of producing hypoimmunogenic wheat. RNAi has been used to make low-gliadin lines. We have presented a range of methods, which combined, enable the seeds from such programs to be efficiently screened. There is still a long way to go in order to make wheat completely safe for coeliacs, as wheat has 100–200,000 ppm gluten and the number of epitopes has to be decreased to the equivalent of 20 ppm. This may mean that several approaches must be combined, and almost certainly, that edited genes from different lines must be combined by crossing and selection within a breeding program. Some of the methods are also suitable for screening during such a breeding program and for determining the safety and quality of the grain produced.

## Author Contributions

This paper was initiated from the General Discussion on a screening pipeline in the Ph.D. thesis of AJ. LG expanded the text and produced Table 1. JS made Figure 1. LG, MS, and JC made Figure 2. MS did the final editing. All other authors were involved in extensive additions and revisions to produce a review of methods. All have read and approved the final version.

## Conflict of Interest

The authors declare that the research was conducted in the absence of any commercial or financial relationships that could be construed as a potential conflict of interest.
